# Evaluation of the Therapeutic Potential of Mesenchymal Stem Cells (MSCs) in Preclinical Models of Autoimmune Diseases

**DOI:** 10.1155/2022/6379161

**Published:** 2022-07-28

**Authors:** Radha Krishan Shandil, Saumya Dhup, Shridhar Narayanan

**Affiliations:** Foundation for Neglected Disease Research (FNDR), 20A, KIADB Industrial Area, Doddaballapur, 561203, Bangalore, Karnataka, India

## Abstract

Autoimmune diseases, chronic in nature, are generally hard to alleviate. Present long-term treatments with available drugs such as steroids, immune-suppressive drugs, or antibodies have several debilitating side effects. Therefore, new treatment options are urgently needed. Stem cells, in general, have the potential to reduce immune-mediated damage through immunomodulation and T cell regulation (T regs) by inhibiting the proliferation of dendritic cells and T and B cells and reducing inflammation through the generation of immunosuppressive biomolecules like interleukin 10 (IL-10), transforming growth factor-*β* (TGF-*β*), nitric oxide (NO), indoleamine 2,3-dioxygenase (IDO), and prostaglandin E2 (PGE2). Many stem cell-based therapeutics have been evaluated in the clinic, but the overall clinical outcomes in terms of efficacy and the longevity of therapeutic benefits seem to be variable and inconsistent with the postulated benefits. This emphasizes a greater need for building robust preclinical models and models that can better predict the clinical translation of stem cell-based therapeutics. Stem cell therapy based on MSCs having the definitive potential to regulate the immune system and control inflammation is emerging as a promising tool for the treatment of autoimmune disorders while promoting tissue regeneration. MSCs, derived from bone marrow, umbilical cord, and adipose tissue, have been shown to be highly immunomodulatory and anti-inflammatory and shown to enhance tissue repair and regeneration in preclinical models as well as in clinical settings. In this article, a review on the status of MSC-based preclinical disease models with emphasis on understanding disease mechanisms in chronic inflammatory disorders caused by exaggerated host immune response in rheumatoid arthritis (RA) and systemic lupus erythematosus (SLE) was carried out. We also emphasized various factors that better predict the translation of stem cell therapeutic outcomes from preclinical disease models to human patients.

## 1. Introduction

Stem cell transplantation is an emerging alternate treatment option for chronic autoimmune disorders. There are dozens of clinically observed autoimmune diseases that affect nearly 5% of the global population [1, 2]. Autoimmune disorders could be systemic or organ-specific. These diseases are caused by “the host immune system attacking itself.” The nature and severity of these disorders depend on the complexity of the immune system and the type of immune response (innate, humoral, or cellular) involved. Major factors underlying autoimmune diseases are environment, lifestyle, genetic disposition, exaggerated immune reactivity, and inflammation and hormones in some cases [3–6]. Autoimmune diseases are typically chronic illnesses, which are difficult to ameliorate and treat. Maintaining a delicate balance between effector and regulatory immune function is required for avoiding autoimmune disorders [5–8]. There are approximately 80 autoimmune diseases known to scientists of which rheumatoid arthritis (RA), systemic lupus erythematosus (SLE), multiple sclerosis (MS), inflammatory bowel syndrome (IBS), Crohn's disease (CD), and type 1 diabetes are well studied both in preclinical models and clinical studies [1, 3, 4, 9, 10].

MSCs are nonhematopoietic, multipotent cells and can differentiate into many cell types, including adipocytes, neuronal cells, and osteoclasts [9, 11, 12]. MSCs can be derived from human bone marrow, adipose tissue, umbilical cord, and dental pulp and can be cultured and expanded to large numbers in vitro to meet the large-scale need for clinical trials and therapy [7, 8, 11, 13–19]. MSCs can home into the injured and inflamed area/capillary beds, where they can sense and communicate with the local cell milieu, collect local data signals, and then intelligently release drug-like substances/growth factors that heal different organs like the liver, lung, bones, knee, and diabetic foot [12, 19, 20]. MSCs have immense immunomodulatory and regenerative capacity, which enables tissue and organ repair and regeneration, a feature distinct from conventional therapeutics. Hence, MSCs have been successfully used to treat inflammation, autoimmune diseases like arthritis and systemic lupus erythematosus, Crohn disease, diabetes, irritable bowel syndrome, the heart, spine injuries, and other organs by transplantation and even infections [11, 12, 19, 21–24].

Conventional therapies for autoimmune diseases have relied on globally dampening the immune responses and arresting the inflammation cycle by using immunosuppressive medications such as steroids, methotrexate, tacrolimus, and antibodies like infliximab and anti-TNF-*α* (Humira) to nonspecifically reduce antibody production or using prostaglandin-cyclo-oxygenase pathway inhibitors [13, 25]. These therapies remain the current standard of care (SOC) and are efficacious in most patients. The caveat is the need for high doses required for longer periods, leaving the patient susceptible to life-threatening opportunistic infections such as tuberculosis in RA patients treated with TNF-*α* antibodies and long-term risk of malignancy [25]. However, many of the treatment modalities currently being investigated lack specificity [13, 25]. While these treatment regimens mitigate the symptoms to a good extent, many benefits are counter balanced by associated toxicity and potentially serious side effects [26, 27]. Thus, developing more target-specific treatment options with reduced risk of systemic immune suppression and reduced toxicity and side effects is the need of the hour [25, 26].

The immunomodulatory properties of MSCs allow downregulation of chronic inflammatory processes. MSCs have been widely studied for immunomodulatory effects using bone marrow, umbilical cord, and adipose tissues to regulate the immune system in experimental models [13, 28–30] and human clinical studies [22–24, 31, 32]. Human clinical trial data show that MSC treatments are well tolerated. Kabat et al. [10] in a comprehensive review summarised results from 914 clinical trials using MSCs, distributed over 14 diseases, including autoimmune diseases. Bone marrow-derived MSCs were most commonly used, and an almost equal number of trials used autologous and allogeneic MSCs, and the median effective dose (MED) suggested was 100 million MSCs/human dose [10]. There are dozens of current ongoing trials reported at http://www.clinicaltrials.gov., encouraging MSC therapeutic development, including for autoimmune diseases. Various studies have demonstrated that adult MSCs can affect the immune T and B cell response by inhibiting the functions of dendritic cells, B cells, and T cells but enhancing the functions of regulatory T cells (T regs) by producing immunoregulatory molecules, thus making them good therapeutic candidates for autoimmune disease treatment [7, 13, 29, 31, 33, 34]. Hence, in this review, we highlight the therapeutic potential of MSC by evaluating their immunomodulatory potential in animal models of rheumatoid arthritis (RA) and systemic lupus erythematosus (SLE) and summarise the underlying immunomodulatory mechanisms specific to these diseases. In addition, the review will also highlight the recent progress made in the clinical translation of MSCs for autoimmune diseases.

## 2. Preclinical Animal Models of Autoimmune Diseases

### 2.1. Rheumatoid Arthritis

Rheumatoid arthritis (RA) is a common chronic autoimmune disease that affects joints and connective tissues leading to chronic joint inflammation, stiffness, pain, and loss of mobility. It is often associated with vascular, metabolic, bone, and psychological comorbidities. Progression of RA is characterized by dysfunction of innate and adaptive immunity, including dysregulated cytokine networks which result in an inflammatory milieu progressively damaging the joint and surrounding tissues [35–37]. Current treatment options are limited, and some patients do not respond well in terms of efficacy, while others may experience detrimental side effects [38]. The most widely used treatment for RA includes corticosteroids [31], nonsteroidal [39] anti-inflammatory drugs (NSAIDs), nonbiologic [40] disease-modifying antirheumatic drugs (DMARDs), and biologic DMARDs [41, 42] like anti-TNF-*α* monoclonal antibodies [42], which are aimed at reducing the symptoms and gradually alleviating the disease pathogenesis.

Since the pathogenesis and clinical presentation of RA are diverse, a significant effort has been made in understanding its aetiology [43, 44], underlying mechanisms, and inflammatory [44, 45] and immunoregulatory pathways [33, 34, 46] to facilitate the development of newer and better therapies [46, 47].

There are nearly one hundred studies published on the development and validation of preclinical disease models of RA, which exhibit promising trends for the clinical application of MSC-based therapeutics. MSC-based studies have demonstrated a reduction of arthritis progression in the majority of preclinical models. Thus, MSC-based therapy may provide relief to patients not responding well to standard of care (SOC) drug-based treatments.

The collagen-induced arthritis model (CIA) in DBA/1 mice and BB, Brown-Norway rats is well validated for studying disease resolution with drugs and MSCs [28, 32, 33, 45–49]. Stem cells injected intravenously or parenterally specifically home into the inflamed arthritic tissues and concomitantly reduce pathogenic cytokines and disease severity scores [8]. The CIA shares many similarities with RA, such as the involvement of Th1 and Th17 cells in disease progression and the presence of autoantibodies; thus, it best represents the systemic immune responses in human RA [46]. Augello et al. injected allogeneic bone-marrow MSCs (BM-MSCs) in DBA/1 mice intraperitoneally (IP). A single injection of these cells significantly reduced inflammatory cytokines, antigen-specific regulatory T cells (Tregs), and disease severity [48].

Similarly, Liu and his colleagues evaluated a single intravenous infusion of human umbilical cord MSCs (hUC-MSCs) on CIA in DBA/1 mice. They again reported an association between decreased proinflammatory cytokines and alleviation of RA symptoms [38]. Liu et al. also demonstrated the therapeutic potential of hUC-MSCs on CIA in DBA/1 mice and showed that upon injecting the cells through IP, a reduction in the severity of arthritis was observed with reduced levels of proinflammatory cytokines and chemokines (TNF-*α*, IL-6, and monocyte chemoattractant protein-1 (MCP1)), induction of Tregs, and increased levels of IL-10 [49]. Similar results were observed when Zhou et al. intravenously (IV) injected human adipose-derived mesenchymal stem cells (hA-MSCs) in a mouse model of CIA. The production of various inflammatory mediators was inhibited upon treatment with hA-MSCs, along with decreased antigen-specific Th1/Th17 cell expansion. Induction in the production of anti-inflammatory cytokine interleukin-10 was also observed.

Moreover, hA-MSCs could induce the generation of antigen-specific Treg cells with the capacity to suppress collagen-specific T cell responses [50]. A robust meta-analysis study [51] that evaluated the utility of MSC therapeutics in preclinical RA models (variables like donor species, tissue of origin, route of administration, and type of transplant—autologous, allogeneic, and xenogeneic) from 1995 to 2019 concluded that MSC therapeutics are good candidates because of their ability to attenuate exacerbated pathogenic immune response in RA and restore the balance between dysregulated proinflammatory and anti-inflammatory cell populations. They postulated that the efficacy of MSC-based therapeutics and amelioration of RA pathogenesis in experimental models could translate to RA in humans [51].

Alternatively, many investigators have employed adjuvant-induced arthritis (AIA) and spontaneous (K/BxN) arthritis models instead of CIA. In an AIA model, MSCs are injected directly into inflamed joints to reduce inflammation [52, 53]. Many studies postulated that higher efficacy with MSCs can be achieved with the infusion of stem cells before disease onset or during the early phase of the disease. Recently, Sampath et al. [54] reported a novel therapeutic combination of placental-derived MSCs (hPMSCs) with stigmasterol, a plant-derived sterol in the monosodium-iodoacetate osteoarthritis (OA) rat model. The authors found beneficial effects on intra-articular administration of hPMSCs with stigmasterol and demonstrated accelerated cartilage repair and regeneration using computerised tomography (micro-CT) and histopathology. This cellular therapy attenuated osteoarthritis lesions with concomitant cartilage repair and regeneration [54].

Numerous studies have been carried out to understand how MSCs decrease proinflammatory cytokines such as TNF-*α* [55, 56] or IL-6 and increase the anti-inflammatory cytokine IL-10, IFN-*γ*-induced protein 10 (IP-10), and/or chemokine receptor 3-alternative (CXCR3) anti-inflammatory cytokine levels in serum and synovium. The precise mechanistic network through which RA is inhibited by MSCs is still being evaluated and emerging. Recently, MSC-derived extracellular vesicles (EVs)/exosomes have emerged as key paracrine messengers that can exert a strong immunomodulatory and therapeutic effect on preclinical models of RA [57].

Though there are convincing preclinical studies on the therapeutic benefits of MSCs in RA, their translation into human RA treatment is an ongoing debate [10, 22]. At present, there is no so-called ideal protocol for MSC-based therapy for RA treatment in human patients [10]. A few clinical studies are available in the public domain (http://www.clinicaltrials.gov.) that evaluate the safety and efficacy of MSC-based therapy in RA, and some are still ongoing [10, 58–61]. So far, these proof-of-concept clinical translation studies have demonstrated promising results in terms of pathology resolution, safety, and efficacy with no to limited adverse events in patients with long histories of RA and particularly in refractory RA patients [59, 61].

### 2.2. Systemic Lupus Erythematosus

Systemic lupus erythematosus (SLE) is a severe autoimmune disease characterized by widespread tissue inflammation and damage to the affected organs. The autoimmune-mediated inflammatory responses in SLE are characterized by the production of autoantibodies/antinuclear antibodies; formation of immune complexes leading to autoantigen accumulation in various tissues including the kidneys, joints, vasculature, and skin; and secretion of proinflammatory cytokines that result in activation of cells of both the innate and adaptive immune systems [62, 63]. Pathogenesis of SLE is multifactorial and is driven by genetics, hormonal factors, immune dysregulation, and immune-mediated inflammatory injury [29, 64].

Current treatment strategies to manage SLE primarily include antimalarials (hydroxychloroquine (HCQ), quinacrine, corticosteroids and nonsteroidal anti-inflammatory drugs (NSAIDs), immunosuppressive drug cyclosporine A (CsA), azathioprine (AZA), methotrexate (MTX), tacrolimus (TAC), and cyclophosphamide (CTX)), mycophenolate mofetil (MMF), and biological agents (rituximab (RTX), belimumab) [24, 65, 66]. However, prolonged use of these drugs leads to toxicity and adverse events, which may promote secondary infections or malignant tumours [24–29, 31–66]. Hence, in severe conditions, alternatives like plasma exchange, high-dose immunoglobulin, or MSC treatment are preferred [67]. Thus, there is an urgent need to develop novel therapeutics for SLE with improved efficacy and reduced toxicity.

MSCs have the potential to regulate the immune system and control inflammation by inhibiting the activation of NF-*κ*B and JACK/STAT and Akt/GSK3*β* signalling pathways to ameliorate SLE lesions [67]. Hence, MSCs have been extensively explored for the treatment of SLE in experimental animals [29, 68] and human patients [2, 8]. MSCs exert immunosuppressive effects through proinflammatory cytokine secretion and inhibiting lymphocyte activation and proliferation. Several studies on the immunomodulatory effects of MSCs on preclinical models of SLE demonstrated the beneficial effect [32, 69–73]. SLE was associated with the activation and proliferation of autoreactive B cells and certain subtypes of T cells [74]. Moreover, deficiency in anti-inflammatory (Treg, Th2) and proinflammatory (Th17, Th1) subsets is one of the crucial factors in the pathogenesis of SLE, leading to tissue inflammation, immune dysfunction, and multiorgan failure [75]. Thus, to understand the mechanism of MSCs as therapeutic for SLE, the best-suited animal model is the Fas mutated MRL/lpr and NZB/W F1 mice, which develop lupus-like syndrome very similar to human SLE and have been widely used to study the mechanism of MSC therapy [76–78]. Jang et al. investigated the effect of hBM-MSCs on the pathogenesis of SLE in female NZB/W mice. They observed that the hBM-MSCs exhibited a protective effect associated with a reduction in autoantibodies, follicular T helper (Tfh) cells, proteinuria, and humoral immune components [79]. A subsequent report by Chang et al. showed that treatment of NZB/W F1 mice with umbilical cord MSCs (hUMSCs) inhibited the pathogenic and inflammatory immune response of SLE and evidently delayed lupus autoimmunity by modulating T cell differentiation and shifting Th1 to Th2 polarization and reducing levels of proinflammatory (TNF-*α*, IL-6, and IL-12) cytokines [29]. In addition, a study conducted by Liu et al. demonstrated that upon xenogeneic transplantation of human placenta-derived MSCs (hP-MSCs) in MRL/lpr mice, a decrease in levels of anti-dsDNA antibodies, NF-*κ*B signalling pathway, expression of TNF-*α*, ICAM-1, plasminogen activator inhibitor-1 (PAI-1), and proteinuria level was observed [80]. Similar results were reported by Zhou et al., whereupon intravenous injection of hBM-MSC in MRL/lpr mice reduced serum levels of anti-dsDNA antibodies, proteinuria, and proliferation of T cells as observed. They also demonstrated a decrease in Th17 cell proportion, IL-17 concentration, and anti-dsDNA antibodies when human Early Embryonic MSCs (hEE-MSCs) were injected in MRL/lpr mice [81]. Park et al. observed similar results with hA-MSC transplantation in Roquin^san/san^ mice. They observed that hA-MSCs markedly ameliorated autoimmunity in a murine model of SLE by decreasing the anti-dsDNA-ICOS+CD44+ follicular helper T cells, Th1, and Th17 and increasing the expression of interleukin-10-producing regulatory B cells [82]. These studies unequivocally demonstrated the immunoregulatory effects of MSCs on T cell populations. Ma et al. also demonstrated downregulation of B cell maturation and differentiation in mice following administration of allogeneic BM-MSCs in MRL/lpr mice [83].

Experimental studies [81–83] in genetically engineered mouse models (MRL/lpr and NZB/W F1) have created compelling evidence that MSC treatment can benefit and ameliorate SLE. These findings supported the exploration of MSC therapy in humans. However, heterogeneous presentation of lupus in humans presents a challenge, and mouse models may not represent a complete spectrum of pathogenesis but only a subset of changes observed in the human population [84]. A comprehensive review of the safety, efficacy, and signal pathways of stem cell therapy of SLE [68] suggested an immense potential for clinical applications. Allogenic MSC transplantation with peripheral blood MSCs in three lupus nephritis patients resulted in decreased serum creatinine levels. Leng et al. [85] assessed the efficacy of autologous peripheral blood-derived MSCs in a 10-year follow-up study in 24 SLE patients and showed a decline in median proteinuria from 4 gm/24 hr to zero levels in follow-up studies after 5 years of transplantation. Liang et al. [86] measured the efficacy of bone marrow MSCs in 15 refractory patients of SLE; 11/15 patients had decreased anti-dsDNA antibodies below baseline during a 12-month follow-up. The major bottleneck in the clinical application of autologous MSC-based therapy in SLE patients was functional abnormalities like cytoskeleton alteration, aberrant cytokine production, impaired phenotype, proliferation, and defective hemopoiesis [83, 87–89]. Due to this malfunctioning, the use of allogenic MSC was preferred for transplantation in SLE patients [30]. Clinical studies with allogeneic MSCs derived from bone marrow and umbilical cord suppressed autoimmunity and restored renal function in patients by reestablishing a balance between Th1- and Th2-related cytokines, decreasing IL-4 levels, and increasing the numbers of Treg cells, TGF-*β*, and IFN-*γ*[30, 32, 88].

#### 2.2.1. Emerging Concepts

Additionally, many new cutting-edge concepts and technologies like exosomes and novel polyherbal drug formulations are emerging that are expected to revolutionize the use of MSC therapeutics in the near future. The use of MSC-secreted exosomes is believed to facilitate communication between cells and their microenvironments by transferring information via their cargo, including the proteins, lipids, and RNAs, gaining momentum as cell-free therapeutics [90]. These exosomes reduced myocardial ischemia/reperfusion injury in mouse models [90]. There are no studies known to our knowledge that have examined this exosomes-based approach from MSCs to treat RA and SLE in animal models or humans that may offer a novel approach in general to treat tissue injury and promote repair in autoimmune disorders [91]. Recently, Renganathan et al. [92] developed a plant-derived ayurvedic polyherbal formulation Dhanwantaram kashayam that regulated lipid metabolism and scavenged oxidative radicals in diabetic rats. This formulation enhanced the proliferation, mobilisation, and homing of stem cells and ameliorated diabetic conditions in experimental rats. These new approaches and formulations need rapid clinical testing and may provide a tangible MSC-based alternative for difficult-to-treat autoimmune diseases. MSC treatments have evoked great expectations, and their wide applicability to many more autoimmune diseases like type 1 diabetes [93] and type 2 diabetes [94] is expected to bring a turning point in modern medicine. Recently, Kotikalapudi et al. in an elegant report published significant success of P-MSC therapy in the control of experimental obesity-associated insulin resistance (IR), regulation of underlying mechanisms, and amelioration of diabetes [94]. They demonstrated homing of intramuscularly injected fluorescent labelled P-MSCs to the visceral region of the adipose tissue by in vivo imaging, activated PI3K-Akt signalling, regulated glucose homeostasis, and insulin sensitivity in dysregulated adipocytes of WNIN/GR-Ob (Ob-T2D) rats. These preclinical findings suggest a potential of P-MSCs in the amelioration and management of type 2 diabetes.

## 3. Conclusions

Stem cell/MSC transplantation treatment is emerging as a rational and alternative therapeutic option for chronic autoimmune disorders like RA, SLE, and type 1 and type 2 diabetes. Undoubtedly, preclinical models of RA and SLE have played a significant role in deciphering underlying immune mechanisms, pathologies, and evaluation of the therapeutic potential of MSCs.

Collagen-induced arthritis models mimic human disease, and the associated markers and events reasonably predict human disease. Though there are convincing preclinical studies on the therapeutic benefits of MSCs in RA, their complete translation into human RA treatment is an ongoing debate. Recent experimental and human studies based on MSC-derived exosome research have yielded exciting results. Further exploration of exosome products in the resolution of RA and regeneration in autoimmune disorders may provide new treatment options.

On the other hand, the pathogenesis of human SLE is complex and varies from patient to patient and even at different times in the same patient. Animal models mimic many of these events but cannot cover the full heterogeneity of human lupus SLE. Nevertheless, animal models have greatly benefited the understanding of complex immunologic and pathologic mechanisms. MSC treatments have been well tolerated in human SLE patients. MSC therapeutics have shown good responses, and findings have translated reasonably to humans as well. Markers like serum autoantibodies (anti-dsDNA, ANA), creatinine, proteinuria, reduction in nephritis and inflammation, and alleviation of SLE lesions seem to well translate into human patients. Unfortunately, large animal models of RA and SLE in canines, sheep, pigs, etc., are lacking that could probably cover the spectrum of pathophysiology better and increase the translation of MSC therapeutics in these chronic ailments.

In summary, human clinical trial data in autoimmune disorders have shown benefit with little or no serious adverse events. There is a linear increase in stem cell human clinical trial registration totalling 914 MSC trials for 14 diseases, including autoimmune diseases, from 2004 to 2018 [10]. There are dozens of currently ongoing clinical trials at http://www.clinicaltrials.gov. which is encouraging for MSC therapeutic development including for many autoimmune diseases.

## Abbreviations

MSCs: Mesenchymal stem cells
BM-MSCs: Bone marrow-derived mesenchymal stem cells
hUC-MSCs: Human umbilical cord-derived mesenchymal stem cells
RA: Rheumatoid arthritis
SLE: Systemic lupus erythematosus
NSAIDs: Nonsteroidal anti-inflammatory drugs
DMARDs: Disease-modifying antirheumatic drugs
SOC: Standard of care
CIA: Collagen-induced arthritis
EVs: Extracellular vesicles
IL: Interleukins
TNF-*α*: Tumour necrosis factor alpha
IFN-*γ*: Interferon gamma
TGF-*β*: Transforming growth factor beta
CXCR3: C-X-C chemokine receptor 3
PGE2: Prostaglandin E2.
